# Safety margins and adaptive capacity of vegetation to climate change

**DOI:** 10.1038/s41598-019-44483-x

**Published:** 2019-06-03

**Authors:** Rachael V. Gallagher, Stuart Allen, Ian J. Wright

**Affiliations:** Department of Biological Sciences, Macquarie University, North Ryde, NSW 2109 Australia

**Keywords:** Climate-change ecology, Macroecology

## Abstract

Vegetation is composed of many individual species whose climatic tolerances can be integrated into spatial analyses of climate change risk. Here, we quantify climate change risk to vegetation at a continental scale by calculating the safety margins for warming and drying (*i*.*e*., tolerance to projected change in temperature and precipitation respectively) across plants sharing 100 km × 100 km grid cells (locations). These safety margins measure how much warmer, or drier, a location could become before its ‘typical’ species exceeds its observed climatic limit. We also analyse the potential adaptive capacity of vegetation to temperature and precipitation change (*i*.*e*., likelihood of *in situ* persistence) using median precipitation and temperature breadth across all species in each location. 47% of vegetation across Australia is potentially at risk from increases in mean annual temperature (MAT) by 2070, with tropical regions most vulnerable. Vegetation at high risk from climate change often also exhibited low adaptive capacity. By contrast, 2% of the continent is at risk from reductions in annual precipitation by 2070. Risk from precipitation change was isolated to the southwest of Western Australia where both the safety margin for drier conditions in the typical species is low, and substantial reductions in MAP are projected.

## Introduction

The composition of vegetation is expected to undergo substantial reassembly in response to anthropogenic climate change^1,2^. Palaeoecological evidence from past climate change events suggests that changes in composition may be rapid^3,4^ and species responses highly idiosyncratic^5^. Studies of contemporary plant communities indicate that changes in composition connected to climate warming are already occurring^6,7^, flagging the need for better predictive tools which estimate risk across spatially explicit aggregates of biodiversity such as assemblages, communities or vegetation types. Predictions of where in the landscape vegetation may be most at risk from the effects of future climate change will be instrumental in directing conservation planning and policy development^8,9^.

Climatic tolerance limits provide valuable information about the potential response of species to changing climates^10–12^. At the species level, approaches for measuring response and adaptive capacity to climate change typically involve detailed experimentation, often including genetic or genomic analyses, to determine fundamental tolerance limits in controlled environments or field settings^13–15^. These approaches are highly informative but cannot be applied practically to entire suites of species occupying whole regions or continents. For hyper-diverse groups – such as plants – scalable approaches which bridge between species-level information on tolerance limits and macroecological patterns are required^16^.

Climatic tolerance limits inferred from the observed niche of species are routinely used to assess the risk to communities and ecosystems from rapid climate change^17–21^. The large-scale digitisation of natural history collections (NHCs) over the last two decades has revolutionised our understanding of the distributional limits of species, particularly in relation to climate. When used appropriately^22^, NHCs are a vital source of information for quantifying climate niches across large cohorts of species^23^. Several climate tolerance indices based on NHC data have been used to assess responses to anthropogenic climate change in marine^19,24,25^ and terrestrial ecosystems^26–29^.

Here, we report an approach that uses observed climatic limits for species derived from NHC data to quantify the safety margin (tolerance), adaptive capacity and risk to continental vegetation from climate change. Using approximately 2.5 million cleaned occurrence records for the Australian flora we calculate observed upper temperature and lower precipitation limits and breadths for 20,608 taxonomically-valid higher plant species. We aggregate these observed limits into metrics of tolerance and adaptive capacity in a set of vegetation units (100 km × 100 km equal area grid cells, hereafter ‘locations’) and combine them with climate projections to assess the likely exposure to future climate change by 2070. We address two questions:

1. How does the risk of climate change to vegetation vary across the landscape and between Australia’s Major Vegetation Groups (MVGs)? Specifically, in which locations does predicted exposure to climate change exceed the safety margin for warming or drying in vegetation? Exposure (*E*) is estimated from projections of future climate for the decade centred on 2070, focusing on two drivers of vegetation distribution at a continental scale – mean annual temperature (MAT; °C) and mean annual precipitation (MAP; mm). Safety margins (*S*) are measured by quantifying how close the climate conditions in a location are to the upper limit of its hypothetical ‘typical’ species (Fig. 1). This metric of climate change tolerance measures how much warmer, or drier, a location could become before its typical species exceeds its observed climatic limit, extending the idea of warming tolerance^11^ to large-scale vegetation communities. Climate change risk is then measured as the difference between exposure and safety margin in a location (*Risk* = *E* – *S*). In locations where *E* > *S*, turnover in species composition may be necessary for vegetation to keep pace with warming or drying conditions. Conversely, a ‘typical’ species in locations where *E* < *S* is expected to be able to withstand projected warming and drying trends. Our focus here on ‘typical’ species allows us to view vegetation as the product of a collection of species responses (while noting that, even where *E* < *S*, at least some species may be adversely affected). We also identify which of Australia’s MVGs may be under most risk from changing climate conditions.

**Figure 1:**
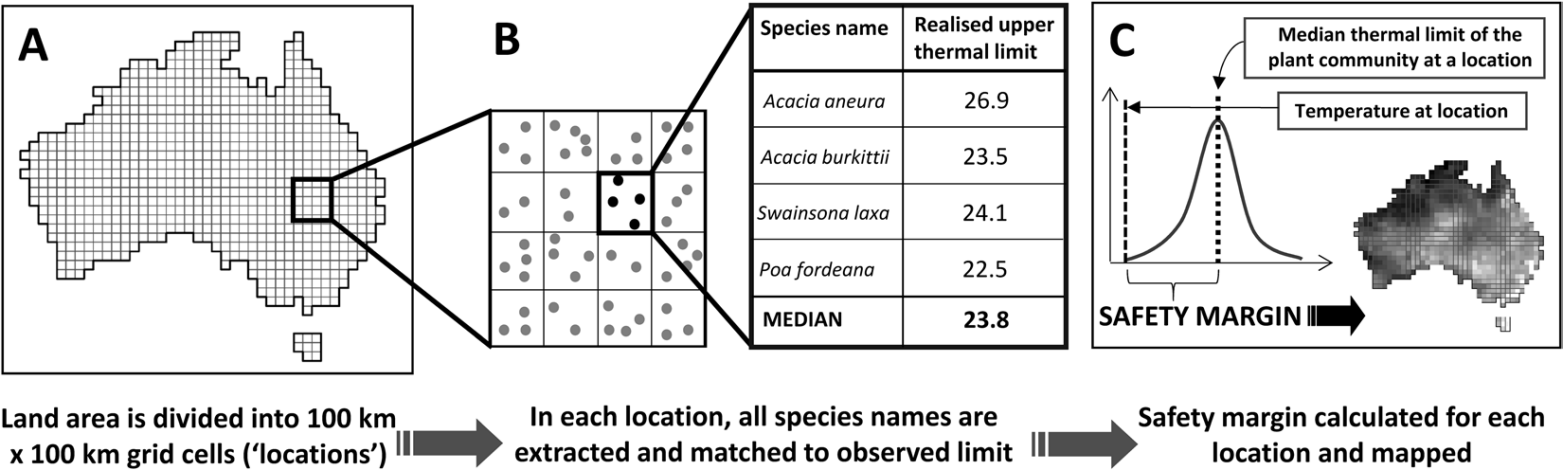
Calculating safety margin at the continental scale. For this example, Australia is divided into a set of 100 km × 100 km, equal area (Albers projection) grid cell locations and intersected with 2,303,015 cleaned species occurrences (latitude and longitude coordinates of vouchered herbarium specimens) (**A**), species lists are extracted for each location and matched to estimates of the upper, observed climate limit for MAT (°). An exemplar community of 4 species is shown here (**B**). In (**C**) a frequency distribution of climatic limits across all species is constructed for each location. The difference between the median of this distribution and the long-term average temperature in the cell estimates the safety margin of the ‘typical’ species to climate warming (MAT example provided here). This climate tolerance metric can then be mapped across the continent.
2. What is the relationship between climate change risk and adaptive capacity in vegetation? We estimate the adaptive capacity of vegetation as the average observed breadth of climate conditions, considered across all co-occurring plant species. We hypothesise that species which span a wider breadth of conditions across their distributional range have a greater capacity to adapt to climate change. By contrast, species with narrow breadths (*i*.*e*., ecological specialists) may need to track conditions which most closely match a particular set of ecological dependencies, limiting their adaptive responses^30,31^. Assessments of individual species ability to adapt to climate change are relatively commonplace and use a variety of techniques from genetics^32^, manipulative experimentation^33,34^ and functional traits^35,36^. However, it remains unclear how species-level adaptive capacity might scale to larger units of biodiversity, such as vegetation types.

## Results

### Climate change risk to Australian vegetation

**Figure 2:**
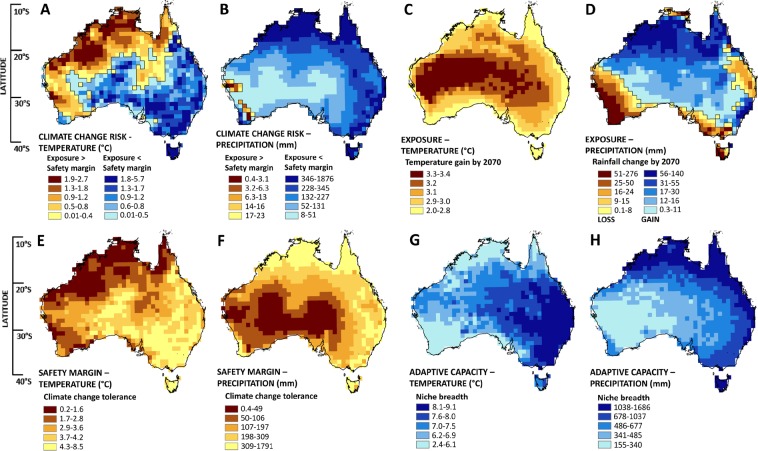
Risk to Australian vegetation from change in mean annual temperature (°C) and precipitation (mm) by 2070 under RCP8.5 (**A**,**B**). Brown regions in A-B indicate regions where exposure to climate change (**C**,**D**) exceeds the safety margin (**E**,**F**) of vegetation based on current MAT and MAP patterns. Exposure is derived from the median projection from five global climate models (ACCESS1.0, CNRM-CM5, HADGEM2-CC, MIROC5, NorESM1-M) centred on the decade 2070 under RCP8.5. Safety margins are based on the difference between observed climate limits and long-term average climate conditions amongst species sharing a 100 km × 100 km grid-cell locations (see Fig. 1). The average climate breadth of species in each location (**G**,**H**) approximates the adaptive capacity of vegetation to climate change (darker blue regions indicating greater adaptive capacity). In all maps, values are coded into five equally-sampled (quantile) bands.

Figure 2 depicts the risk (A-B), exposure (C-D), safety margin (E-F) and potential adaptive capacity (G-H) of vegetation to projected rates of climate change by 2070 under RCP 8.5. Brown regions in (A) and (B) indicate locations where exposure to climate change exceeds the safety margin of vegetation based on current MAT and MAP, and *vice versa* for blue locations. Values for each location are available in Supplementary Table S2.

#### Exposure

As a percentage of land area, 100% of Australian vegetation is exposed to increases in MAT and 26% to decreases in MAP by the decade centred on 2070 (Fig. 2C,D). The median projection across five GCMs shows exposure to MAT increase is likely to be greatest in the central inland regions of the continent (3.3–3.4 °C) however substantial warming is also projected in coastal regions (2.0–2.8 °C). Exposure to changing precipitation patterns also varies markedly across the continent. Substantial decreases in MAP (dark brown colours, Fig. 2D) are broadly predicted for coastal regions in the south and for small areas in the north (coastal Queensland and Northern Territory). Substantial increases in MAP are predicted for vast areas of northern Australia and for coastal New South Wales, in the east.

#### Safety margins

Safety margins for increase in MAT in the vegetation of Australia range between 0.2–8.5 °C. Vegetation across 8% of Australia has a safety margin of <1 °C, and 26% a safety margin of <2 °C (Fig. 2E). Areas with the smallest safety margins are clustered in the tropics – particularly in the north-west of the continent – and in the upper part of the southwest floristic region of Western Australia. In these locations, based on the observed climate limits of their typical species, vegetation has a relatively small safety margin for adjusting to projected increases in temperature. The vegetation with the most tolerance (largest safety margin) to temperature change occurs across the south-east corner of Australia, including the majority of Tasmania and extending into coastal parts of Queensland and the central inland. Many of these regions with large safety margins are characterised by relatively high topographic relief (>1000 m elevation; *e*.*g*., the Australian Alps in the south east, the Border Ranges in northern NSW, the Flinders Ranges in South Australia, Tasmania, Atherton Tablelands in far north Queensland).

Across Australia, safety margins for decreases in MAP range between 0.4–1791 mm and 38% of vegetation has safety margins which are smaller than a ≤ 100 mm decrease in MAP (Fig. 2F). That is, the typical species across 38% of the continent can tolerate a reduction of precipitation of 100 mm or less before exceeding its observed climate limit. MAP itself varies widely across the continent so there is also merit in considering proportional reductions in precipitation. When safety margins are calculated relative to a proportional 10% reduction in MAP at each location, 18% of continental vegetation exceeds its observed lower limit, increasing to 95% of the continent under a 50% reduction in MAP.

The smallest safety margins for reductions in MAP are concentrated across the desert regions of the central western inland (*i*.*e*., dark brown regions; Fig. 2F). Conversely, vegetation which is relatively tolerant to MAP change is clustered in regions characterised by high annual precipitation, such as the tropical regions of far north Queensland (*i*.*e*., light-yellow region in coastal areas <20°S).

**Figure 3:**
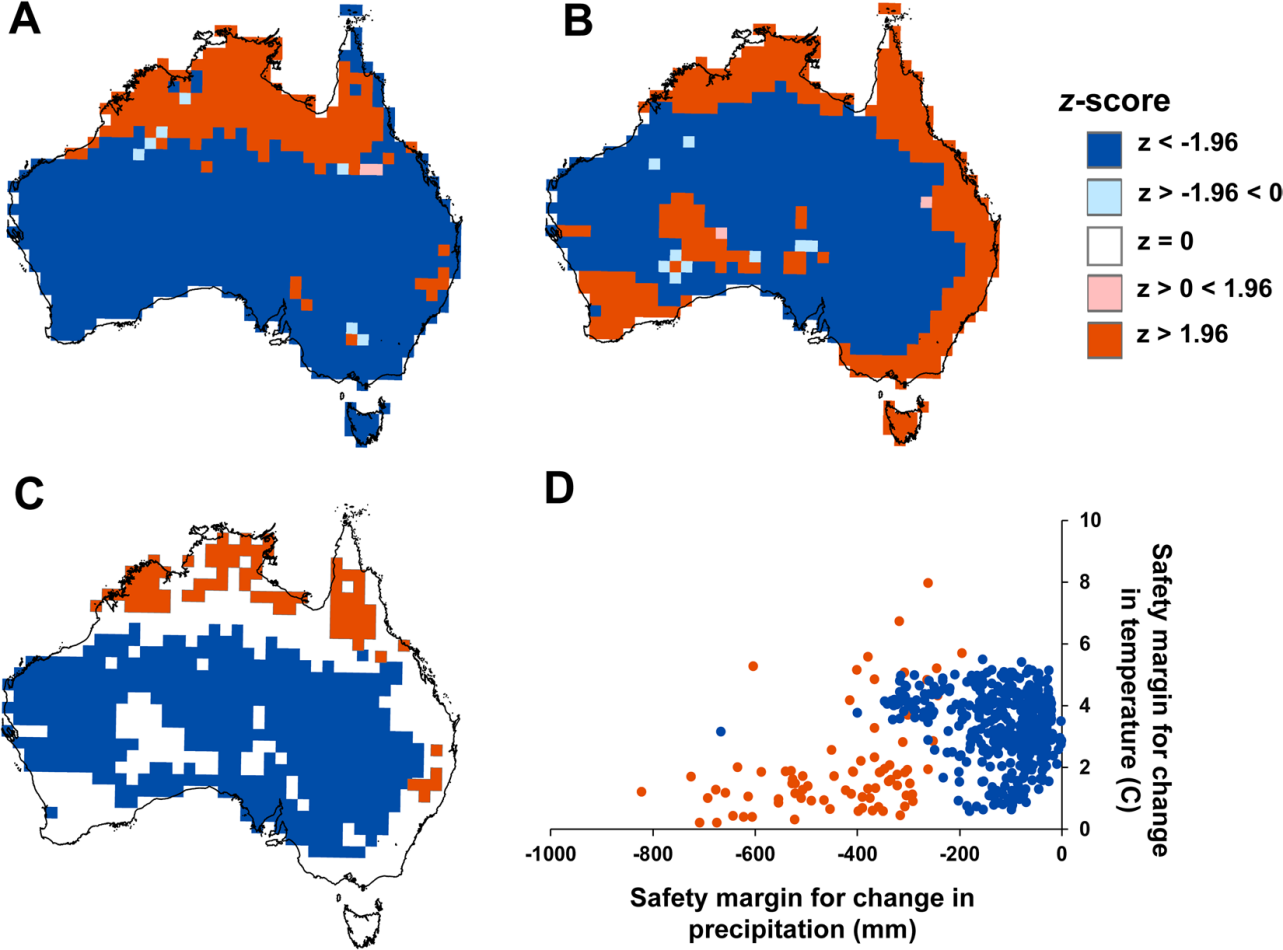
Standardised scores of the safety margins of Australian vegetation to changes in MAP (**A**) and MAT (**B**). Maps depict the deviation of the observed safety margin from random expectation (*z*-score) under a null model using 1000 reassignments of observed niche limits. Dark blue and red represent locations where safety margins are lower or higher than expected, respectively, given observed species richness. Regions which have vegetation with significantly larger (*i*.*e*., *z*-score ≥ 1.96), or smaller (*i*.*e*., *z*-score ≤ 1.96), safety margins relative to chance for both MAT and MAP are depicted in (**C**). The relationship between observed safety margins for MAP and MAT is shown in (**D**).

As MAT and MAP safety margins are measured in differing units, standardised *z*-scores were also calculated for each location to allow comparison between climate variables (Fig. 3A,B). This null modelling indicated that the observed safety margins across 21% (MAT) and 34% (MAP) of vegetation were larger than expected under random expectation (dark red locations; Fig. 3A,B). In these locations, after comparing the observed and expected values, the comparative chance of detecting the observed safety margin was <5% (i.e. *z*-score =≤1.96, or ≥1.96).

Vegetation with significantly larger safety margins relative to chance for both MAT and MAP (*i*.*e*., *z*-score ≥1.96) occur across 11% of Australia (red locations in Fig. 3C). Conversely, vegetation with significantly smaller safety margins than expected for both climate variables (*i*.*e*., *z*-score ≤ 1.96) are found across the majority (60%) of Australia (blue locations in Fig. 3C). In these regions, most locations have safety margins for reductions in MAP of <200 mm and for temperature change between 2–4 °C (Fig. 3D).

#### Climate change risk

Locations at risk are those where exposure to climate change exceeds the safety margin of the typical species (*Risk* = *E* - *S*; brown locations in Fig. 2A,B). Using this metric, vegetation across 47% of Australia is at risk from MAT increase by 2070. As the magnitude of exposure to MAT change is relatively uniform across the continent (2–3.4 °C; Fig. 2C) patterns of risk are largely driven by the safety margin (tolerance) of vegetation to climate change. By contrast, only 2% of the continent is at risk from reductions in MAP by 2070 (Fig. 2B). Precipitation vulnerability is isolated to the southwest of Western Australia in places where both the safety margin for drier conditions in the typical species is low (low tolerance) and substantial reductions in MAP are projected (high exposure). Across the five GCMs analysed, MAP is projected to decrease by between 25–276 mm by 2070 in this region.

#### Adaptive capacity

Based on estimates of the niche breadth of a typical species, the adaptive capacity of vegetation to MAT increase and MAP decrease under climate change ranges between 2.4–9.1 °C and 155–1686 mm, respectively (Fig. 2G,H). Higher values indicate vegetation is adapted to a wider breadth of MAT and MAP and assumed to have the potential to adapt *in situ* to climate change. Vegetation with relatively high adaptive capacity for temperature change is mainly located in the south-east of the continent and the south-west of Western Australia has the lowest adaptive capacity to climate change based on our estimates of the niche breadth of a typical species. Desert regions of Western Australia have the lowest adaptive capacity for MAP change (light blue regions in Fig. 2H), though precipitation is already low in these regions and plants exhibit many adaptations to arid conditions.

**Figure 4:**
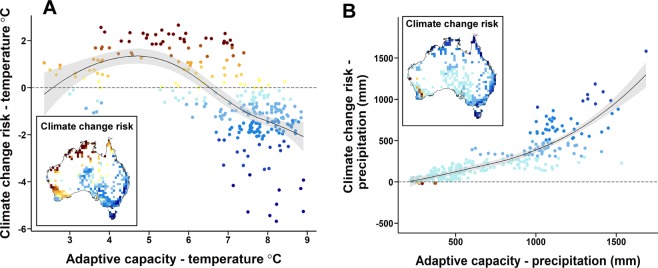
Relationship between risk and adaptive capacity to climate change in Australian vegetation. Risk from changes in MAT (°C; **A**) and MAP (mm; **B**) by 2070 under RCP8.5 is calculated as the difference between exposure to climate change and the safety margin to warming, or drying, of a typical species in each location. Adaptive capacity is calculated as the climatic breadth across the range of a typical species in each location. ‘Typical’ species are represented by the median response of all species present at a location. Replicates in (**A**,**B**) are locations (grid cells) from across Australia. Only locations with high herbarium sampling completeness (ratio of observed to expected richness from Chao1 estimator ≥0.7) were included as replicates in statistical analyses (*n* = 388 cells; 49% of Australian land area). The black line and grey fill depict the LOESS regression line and its confidence intervals. Note that for MAT, at risk locations have positive values on the *y*-axis whereas for MAP values are negative. This is because risk values summarise a tolerance deficit for the typical species in terms of increasing temperature or decreasing precipitation.

To test hypotheses about relationships between risk and adaptive capacity we used a subset of locations (*n* = 388 grid cells) where completeness of herbarium specimen sampling was ≥0.7 based on the ratio of observed to expected richness derived from the Chao1 estimator (inset maps Fig. 4; Supplementary Figure S1 and Supplementary Table S2). These locations cover 49% of the Australian land area and span all biomes present (Bureau of Meteorology, 2006).

There was a significant curvilinear relationship between adaptive capacity and risk of MAT change in Australian vegetation (R^2^ = 0.42, F _(2,283)_ = 100.8, *p* < 0.001; Fig. 4A). Most locations (69%) with low risk under temperature increases also had high adaptive capacity for this variable (*i*.*e*., median climate breadths >7.5 °C). We also found a significant curvilinear relationship between risk from MAP change and potential adaptive capacity, whereby the most vulnerable locations were those characterised by species with narrow climate breadths for precipitation (R^2^ = 0.70, F _(2,283)_ = 326.9, *p* < 0.001; Fig. 4B).

#### Climate change risk to Major Vegetation Groups (MVGs)

**Figure 5:**
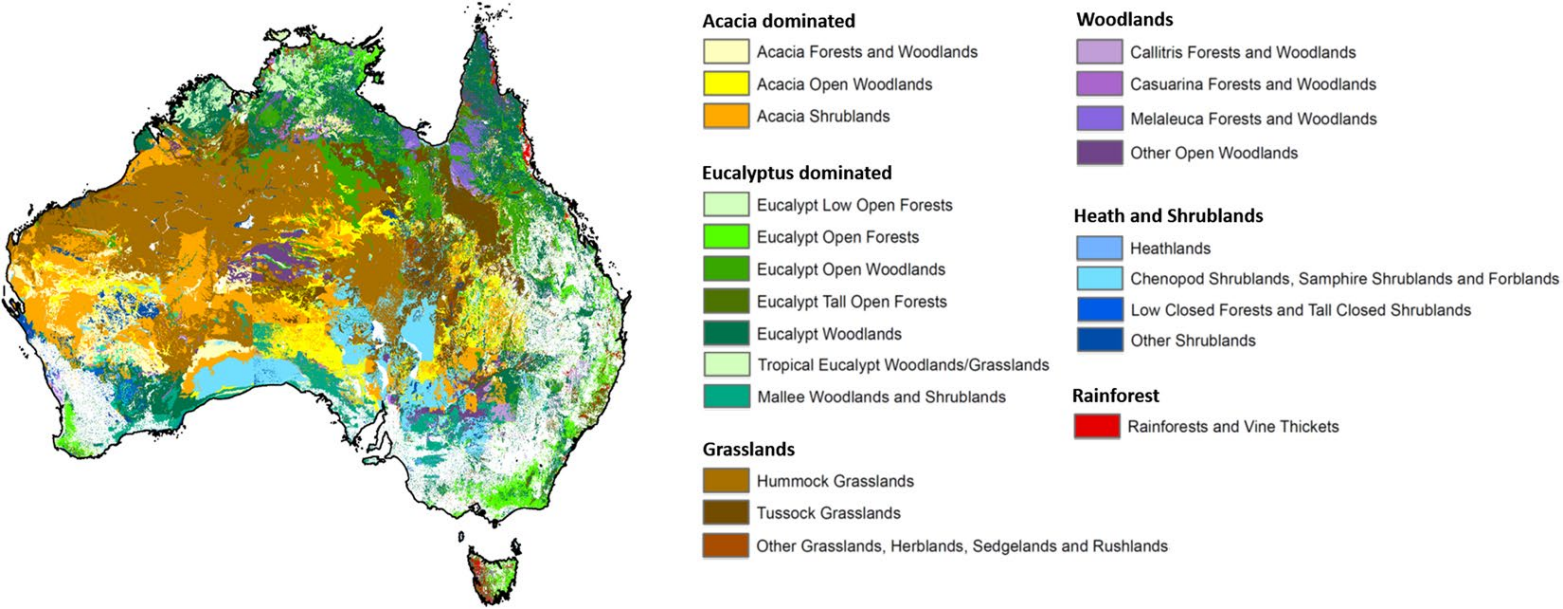
Twenty-two Australian Major Vegetation Groups (MVGs). MVGs have been grouped into simplified vegetation units (*e*.*g*., Acacia dominated) which are coloured in similar hues. White map units denote areas mapped as the MVGs excluded from our analyses (Inland aquatic - freshwater, salt lakes, lagoons; Cleared, non-native vegetation, buildings; Naturally bare - sand, rock, claypan, mudflat; Mangroves; Regrowth, modified native vegetation; Sea and estuaries; Unclassified forest; Unclassified native vegetation; Unknown/no data). Source: National Vegetation Information System (version 4.2).

Of the 22 MVGs analysed (Fig. 5), 21 occur in areas deemed at risk from increases in MAT by 2070, and 13 in areas at risk from MAP decreases (Table 1). ‘Tropical Eucalypt Woodlands/Grasslands’ are most at risk, with 98% of their extent occurring in areas where exposure to climate change exceeds the safety margin for warming of the typical species present. Other at-risk MVGs include: ‘Melaleuca Forests and Woodlands’ (91% of extent at risk); ‘Low Closed Forests and Tall Closed Shrublands’ (78%); and ‘Hummock Grasslands’ (77%). Both ‘Melaleuca Forests and Woodlands’ and ‘Tropical Eucalypt Woodlands/Grasslands’ are restricted to the tropical north of Australia and are relatively small in overall extent across Australia (Area (%); Table 1). Similarly, ‘Low Closed Forests and Tall Closed Shrublands’ which are found in Western Australia have a very small overall extent (0.2% of Australia), 78% of which falls in areas of climate change risk.

**Table 1:** Climate change risk analysis of Australia’s Major Vegetation Groups (MVG).

MVG code	Vegetation Unit (MVG name)	Simplified Vegetation Unit	Area (% Australia)	% of MVG under climate change risk (MAT)	% MVG under climate change risk (MAP)
6	Acacia Forests and Woodlands	Acacia dominated	5.5	38.5	3.2
13	Acacia Open Woodlands	Acacia dominated	4.2	20.6	0.4
16	Acacia Shrublands	Acacia dominated	11.5	49.6	7.2
4	Eucalypt Low Open Forests	Eucalyptus dominated	0.1	6.3	0.4
3	Eucalypt Open Forests	Eucalyptus dominated	3.7	22.9	0
11	Eucalypt Open Woodlands	Eucalyptus dominated	6.2	56.7	0.1
2	Eucalypt Tall Open Forests	Eucalyptus dominated	0.5	0	0
5	Eucalypt Woodlands	Eucalyptus dominated	12.1	53.5	0.3
14	Mallee Woodlands and Shrublands	Eucalyptus dominated	3.7	20.8	1.6
12	Tropical Eucalypt Woodlands/Grasslands	Eucalyptus dominated	1.5	97.8	0
20	Hummock Grasslands	Grasslands	18.5	76.9	0
19	Tussock Grasslands	Grasslands	7.1	53.5	0
21	Other Grasslands, Herblands, Sedgelands and Rushlands	Grasslands	0.9	50.9	0
22	Chenopod Shrublands, Samphire Shrublands and Forblands	Heath and Shrublands	5.9	15.5	0.3
18	Heathlands	Heath and Shrublands	0.1	42.4	0.9
15	Low Closed Forests and Tall Closed Shrublands	Heath and Shrublands	0.2	77.6	3.7
17	Other Shrublands	Heath and Shrublands	1.7	49.6	5.5
1	Rainforests and Vine Thickets	Rainforest	0.5	22.0	0
7	Callitris Forests and Woodlands	Woodlands	0.4	2.1	0
8	Casuarina Forests and Woodlands	Woodlands	2.0	15.2	0.1
9	Melaleuca Forests and Woodlands	Woodlands	1.3	90.8	0
10	Other Forests and Woodlands	Woodlands	1.0	62.6	0.3

The total spatial extent of MVGs across Australia (Area % Australia) and the amount of the MVG within areas of climate change risk under projected MAT increases and MAP decreases are provided. Simplified Vegetation Units correspond to Fig. 5. MVG codes and names are those assigned by the National Vegetation Inventory System (v. 4.2).

Projected changes in MAP relative to vegetation safety margins place several vegetation communities at risk in the south-west of Western Australia, though the amount of vegetation affected may be small relative to its overall extent. For instance, ‘Low Closed Forests and Tall Closed Shrublands’ are at risk, but only 3.7% of their overall extent is threatened. Various Acacia-dominated MVGs (Fig. 5; Table 1) are also at risk from decreases in MAP, however the total extent of the MVGs affected is small (*e*.*g*., 7.2% of ‘Acacia Shrublands’; 3% of ‘Acacia Forests and Woodlands’).

## Discussion

Vegetation across almost half of Australia (47%) may be at risk from projected increases in MAT based on spatial analysis of exposure and safety margins for temperature climate change by 2070. Risk from MAT change is most acute in the northern, tropical regions of the continent and across most of the state of Western Australia (*i*.*e*., brown cells in Fig. 2A). In these regions, projected exposure to MAT change by 2070 exceeds the inherent safety margin, or tolerance, to warming of the typical species that characterises this vegetation (measured as the difference between the median, upper temperature limit across all species present, and the long-term MAT in each location (Figs 1 and 2E,F)). The adaptive capacity of vegetation to tolerate changes in MAT is predicted to be highest in regions of low climate change risk (Fig. 4). By contrast, at-risk regions with low adaptive capacity under warming conditions – such as Tropical Eucalypt Savannas in the north, and the Southwest Australian Floristic Region (SWAFR) of Western Australia – may be important targets for intensive conservation management, particularly where land-use is rapidly changing. The SWAFR also contains the only vegetation identified as at -risk from projected reductions in MAP by 2070. Across this vulnerable vegetation – which covers 2% of the continent (170,000 km2) – MAP is consistently projected to decrease by 2070 across the five GCMs assessed and the safety margin for drying of a typical species is larger than these projected reductions. When directly compared using *z*-scores, safety margins for both temperature and precipitation are significantly larger than expected by chance across the tropical regions of the continent (red locations; Fig. 3C); smaller margins than expected are found across large areas of the arid zone (blue locations; Fig. 3C), increasing the likelihood of climate related impacts on vegetation composition in these regions due to the interactive effects of changes in temperature and precipitation.

Our analysis constrains the upper climate limit of all species assessed to the highest MAT across a study region (*i*.*e*., 29.4 °C at a 5-arc minute resolution in our Australian example). This means that no species can have an observed climate limit higher, or lower, than the highest value of MAT or MAP recorded across Australia. Of the 20,608 species assessed here, 151 (0.7%) have upper observed climate limits for MAT occurring at this 29.4 °C threshold, and a further 1,147 species have upper limits within 1 °C of this threshold. These 151 species are important targets for physiological/experimental research, particularly those which are dominant and widespread such as *Eucalyptus tetrodonta* and *E*. *camaldulensis*, to characterise their temperature tolerances and adaptive capacity under climate change. Increases in mortality in *E*. *tetrodonta* in the Victoria River regions of the Northern Territory have been attributed to drought-related die-back due to localised dry-season water stress^37^ and increasing incidence of heat wave events may challenge species which currently occupy the warmest locations across Australia^38^.

Our approach to continental scale analysis of vegetation offers a biogeographic perspective on the exposure, safety margin, risk and potential adaptive capacity of the Australian flora. This analysis can identify regions and species of greatest concern for conservation planning (*e*.*g*., MVGs contained largely within at-risk areas, such as ‘Tropical Eucalypt Woodlands/Grasslands’ (Table 1); or, areas with smaller safety margins for MAT and MAP under climate change than expected by chance (Fig. 3C). Similarly, data on observed climate limits can be used to identify species to target for physiological experimentation (e.g., the 151 species with observed climate limits at Australia’s highest MAT). Currently, detailed physiological experimentation to quantify the response of Australian vegetation to climate change is either highly site-based (*e*.*g*., the EucFACE and OzFACE facilities) or is restricted to examining a modest subset of species (*e*.*g*.^33,39^). Our approach complements the use of species distribution models^40–42^ and dynamic global vegetation models^43^ to assess climate change risk to Australian vegetation. Climate change vulnerability analyses have been conducted at smaller scales for singular Australian plant taxa or communities^44–46^ and at similar continental scale to our analysis for birds^26^.

Projected decreases in MAP exceed the drying tolerance of vegetation in south-west of Western Australia. In the SWAFR, vegetation in regions as at risk from MAP change in our analysis are already undergoing large-scale die-back events attributed, in part, to increased incidence of hot and dry conditions^47,48^. Similarly, Western Australia’s northern Jarrah forests, species which occur on rocky soils with low water holding capacity are known to suffer high rates of dieback during extreme dry years^48^. Our estimates of precipitation effects on risk are presumably very conservative since temperature and precipitation interact strongly. For instance, drier conditions can result even in the absence of a projected decrease in precipitation because increased MAT is accompanied by increased evaporation from the land surface, resulting in lower available soil moisture for a given precipitation. That said, there will be some tendency for higher atmospheric CO_2_ to ameliorate the physiological effects of reduced precipitation, through its positive effect on photosynthetic water use efficiency^49^.

Despite the high vulnerability of the SWAFR to changes in rainfall, the typical species in vegetation across most of Australia has relatively high ability to withstand reductions in MAP projected for the decade 2070. That is, projected reductions in MAP are far smaller than the safety margin for drying of the typical species across 98% of the continent. In one sense this simply reflects the projections of GCMS which show that a relatively small area of Australia is exposed to substantial MAP decrease by 2070 (*i*.*e*., only 5% of the continent is in the upper quantile for precipitation reductions by 2070 – dark brown regions of Fig. 2D). But in addition, this low risk to MAP change may be the result of the inherent unpredictability of precipitation^50^ and the adaptations of many Australian species to variability. For instance, our safety margin metric shows that, relative to average precipitation totals between 1950–2000, the average species in 95% of Australian vegetation may tolerate a reduction of between 10% and 69% of current precipitation amount before exceeding its observed climate limit. This is a conservative measure given that the predicted higher temperatures across much of the continent will lead to higher rates of evaporation, hence generally less plant-available water for a given MAP.

### Considerations and caveats

Abiotic factors not considered in our analysis can modify plant exposure to temperature and precipitation change at local scales, such as microtopography and aspect^51,52^ and soil conditions (*e*.*g*., soil depth, soil water holding capacity, and the mosaic of run-off/run-on zones created by topographic variation^53^). Where species composition is known to change rapidly over relatively short altitudinal gradients (*e*.*g*., the Australian Alps, and the Wet Tropics Bioregion) the response of the typical species may be more difficult to reasonably characterise using the median safety margin (and median climate limits). In these locations, vulnerability assessments of individual plant communities or species will offer more nuanced insights on the threat of climate change^54^. However, we caution against solely assessing rare species (narrow-ranged endemics) in vulnerability analyses at the expense of their more common counterparts^55^.

Our analysis was limited to the use of estimates of the observed climate niche of species across their historical distribution, as opposed to using assessments of the highest MAT or lowest MAP under which plant function can be maintained (*i*.*e*., species’ fundamental niche limits of Grinnell^56^). We acknowledge that fundamental limits may not be bounded by conditions experienced across the range; species may not be physiologically intolerant of cooler or warmer, drier or wetter conditions not encountered across their historical distribution. That said, a recent meta-analysis of eleven reciprocal transplant experiments showed strong support for the idea that geographic range limits closely approximate niche limits^57^. Our analysis captures which species have been recorded at least once at a location, though as herbarium collections accumulate over time current day composition may differ. Similarly, herbarium collections offer no accompanying data on species abundance and this may affect estimates of safety margins.

Gathering data on fundamental limits is time-intensive and therefore necessarily limited to a small subset of (presumably) representative species. We generously estimate that c.1% of the flora of Australia (approximately 200 species) have had fundamental tolerance limits to temperature and precipitation characterised via experimentation, and that these species are heavily biased toward a few species in the genera *Eucalyptus* and *Acacia*. Although these genera are dominant in many landscapes, they represent a relatively modest fraction (c. 10%) of the plant diversity in Australia, which encompasses c. 20,000 species from >200 families.

Biotic interactions (*e*.*g*., inter-specific competition or facilitation, herbivory) and barriers to dispersal which prevent occupation of locations where species are physiologically tolerant may also lead to underestimations of the niche limits of species^58,59^. Finding a balance between taxonomic coverage and experimental determination of responses when assessing vulnerability to climate change will remain a challenge until more comprehensive data accumulates on species fundamental limits.

## Methods

### Cleaning species occurrence data

Digitised collection records from 4,312,554 vouchered herbarium specimens in the *Australian Virtual Herbarium* (AVH) were accessed via the *Atlas of Living Australia* (ALA) application programming interface http://api.ala.org.au/. Analyses were limited to terrestrial, higher-plant taxa native to Australia’s mainland states and Tasmania (excluding offshore island territories, such as Norfolk, Christmas, Lord Howe, Macquarie and Heard Islands).

We used the Australian Plant Census (APC) as our taxonomic authority^60^; available here: https://biodiversity.org.au/nsl/services/APC). The APC provides accepted scientific names for native and naturalised plant species in Australia. However, the APC does not classify families based on higher groupings; *i*.*e*., higher plants (angiosperms, gymnosperms) or lower plants (ferns, hornworts, liverworts). To do this, we used the R package *taxonlookup*^61^ to assign families to higher and lower plant groupings and limited our analyses to higher-plant families (*n* = 226).

Filters were applied to limit AVH records to valid spatial and taxonomic occurrences. This resulted in 2,303,015 valid occurrences across 20,608 species (mean and median of records per species are 112 and 45 respectively). Collection records were eliminated where they met one of the following criteria: (1) no valid species name in the APC, including being known only by an unratified name or hybrid designation (8.3% of raw data, *n* = 360,684 records), (2) lack of geo-referenced coordinates, or coordinates which fall outside the mainland and Tasmanian Australian terrestrial administrative boundaries (9.9%, *n* = 427,563 records), (3) flagged as ‘suspected outliers’ by the ALA using a reverse jack-knife procedure assessing similarity of occurrences in environmental space (4.4%, *n* = 191,131 records), (4) cultivated origin, either flagged as such in the AVH or containing the search terms cultivat* and/or garden in a free-text search of the collection record (1.9%, *n* = 82,290 records), (5) being of non-native origin, identified by cross-checking to an exotic species checklist for Australia^62^ or to a list of species naturalised in at least one state of Australia in the APC (18.7%, *n* = 805,960 records), (6) being an observation other than a preserved specimen (0.3%, n = 14,447 records. Note that species often met more than one of these criteria and Fig. S2 provides a histogram of the year of collection of all specimens.

We retained only one unique record for each combination of species name, latitude, longitude, month, year, and collector name in the data set to remove potential duplicate specimens (9.2%; *n* = 400,611 records) of the same collection event submitted to the AVH from multiple state herbaria.

### Sampling completeness

Sampling effort in herbarium collections varies spatially, often in concert with human settlement and infrastructure^63^. To minimise the effect of potentially inadequate sampling on vulnerability analyses we excluded 402 locations (100 km × 100 km grid cells) with low sampling completeness (<0.7) from our analyses. Completeness was estimated from the ratio of observed to expected species richness, where expected richness was calculated from occurrence records using a bias-corrected Chao1 estimator^64,65^ in R (R Core Team, 2014) using package *vegan 2*.*3–4*^66^.

### Quantification of species’ temperature and precipitation limits

We matched cleaned occurrence records for each species to gridded long-term average climate conditions to identify the highest MAT and lowest MAP at which the species occurs. Climate data were matched at a 5 arc-minute resolution (~8 km) to approximate the average spatial accuracy of herbarium collections. The 98^th^ percentile of MAT and 2^nd^ percentile of MAP were taken as the upper and lower observed climatic limits for each species, respectively. Climate data were accessed from Worldclim^67^. All analyses were performed using the packages *raster*^68^, *rgdal*^69^ and *sf*^70^. Observed species limits are available as Supplementary Table S1.

Observed climatic envelopes derived from occurrence data potentially underestimate the fundamental climatic tolerances of species^22,71^. We used two approaches to partially address this issue. First, we tested the effect of combining our cleaned occurrence records with cultivated occurrences from 122 botanic garden inventories across Australia when estimating the observed upper temperature limits of species. This allowed us to the assess whether climate limits from natural history collections routinely underestimate the climate limits of the species and, therefore, affect the outcomes of our analyses^71^. Second, we assessed whether the climatic limits of a suite of tropical species which occur in higher latitudes outside Australia are truncated by abutting the continental land limits of Australia. If widespread, this truncation could decrease the accuracy of estimates of the climatic limits of species.

Cultivated records from botanic gardens inventories published in Bush *et al*. (2018) were available for 6,159 species in our dataset (30%). We matched the locations (latitude and longitude coordinates) of each botanic garden and species combination to gridded climate data as described for all other occurrence records. We then counted how many species had upper climate limits from cultivated records which exceeded estimates from native range occurrences. It was not possible to calculate an analogous metric from cultivated records for MAP because of the confounding effect of irrigation in botanic gardens on plant performance. The effect of irrigation is also likely to increase the ability of cultivated plants to tolerate warmer conditions; additional water increases the potential for evaporative cooling to maintain favourable leaf temperatures for photosynthesis^39,72^.

Only 17% of species for which data were available (*n* = 1042 of 6159) had climatic limits for MAT which were higher when derived from species occurrences in botanic gardens (the potential niche^71^) than when derived from vouchered herbarium specimens. These 1,042 species account for only 5% of the species assessed in our study (*n* = 20,608) and the effect of these species on mapped patterns of safety margins at the continental scale was considered to be negligible. Henceforth, we report findings derived from vouchered herbarium specimens only.

To assess the potential truncation of the observed limits of species due to distributions being bounded by continental land limits we randomly selected 10% of species endemic to the tropical region of Australia (*n* = 370 species) and collated their occurrence data from tropical land areas north of Australia (*e*.*g*., Papua New Guinea, Indonesia, Malaysia, Solomon Islands) from the Global Biodiversity Information Facility (www.gbif.org). Vouchered herbarium occurrences were available from these regions for 42 species and their upper MAT limits were extracted following methods described for Australian occurrences. We then calculated the difference between climatic limits derived from inside and outside Australia. The extent of truncation of climatic limits was relatively small, with most species (81%; *n* = 34 species) having *lower* limits for MAT in locations outside Australia. For the remaining species (*n* = 8) upper MAT limits were <1° higher outside Australia (0.3–0.9 °C). Therefore, we assume that upper observed climate limits for temperature are rarely truncated by continental land limits in this study and that any truncation will be negligible when calculating the median safety margin of a typical species in each location across Australia (see Climate change risk predictions below).

### Climate change risk predictions

The risk to vegetation from projected climate change in each location was quantified from two components – safety margin and exposure^73^.

Safety margin was calculated for each location as the difference between the median observed climatic limit across all species present and the long-term average climate conditions in the location (Fig. 1). This measure captures how much warmer, or drier, a location could become before its hypothetical typical species exceeds the observed climatic limit.

We also created standardised scores (*z*-scores) for the safety margin at each location for MAT and MAP to allow for comparison of patterns between climate variables which are measured in differing units (°C, mm). In each location, we performed 1000 random draws from the observed climate limits of all Australian species, constraining the species pool to those with an upper (MAT) or lower (MAP) limit which was lower or higher, respectively, than the long-term climate in the cell. This randomisation procedure produced an expected safety margin in each location which was compared to observed values to calculate *z*-scores.

Exposure to climate change in each location was derived from anomalies between current and future projected climate conditions. To calculate anomalies for MAT and MAP, current long-term average climate conditions were subtracted from downscaled projections of future climate from five global circulation models (GCMs) in the Coupled Model Inter-comparison Project Phase 5 for the decade centred on 2070 accessed from www.worldclim.org. The median anomaly across the five GCMs (ACCESS1.0, CNRM-CM5, HADGEM2-CC, MIROC5, NorESM1-M) was calculated and aggregated to a 100 km × 100 km equal-area grid. These five GCMs were chosen for their high skill-scores in modeling various Australian climatic phenomena (*e*.*g*., El Niño-Southern Oscillation, historical climate, regional variability)^74^. GCM projections were based on a comparatively high atmospheric greenhouse gas concentration pathway (Representative Concentration Pathway (RCP) 8.5; *c*. 800 ppm of CO_2_ by 2070) and multiple models were used to capture known differences in the assumptions which underpin the production of future climate scenarios^75^.

Climate change risk to vegetation in each location was calculated as the difference between the exposure and safety margin (*Risk* = *E* - *S*). Where *E* > *S*, vegetation is predicted to be at risk from climate change.

### Adaptive capacity predictions

We quantified the adaptive capacity of vegetation at each location by aggregating climate breadths calculated for each species. We assumed that a wider median climate breadth across all species at a location indicates a greater adaptive capacity to changing climate for the typical species in the vegetation^30,76^. For each species, climate breadth was quantified as the difference between the 2^nd^ and 98^th^ percentiles of MAT and MAP conditions, respectively, experienced across the range of each species.

We tested for a relationship between climate change risk and adaptive capacity across Australian vegetation using ordinary least squares regression (α = 0.05). Where high climate change risk and low adaptive capacity are coupled together in the landscape, the likelihood of vegetation change may increase.

### Climate change risk to Major Vegetation Groups (MVGs)

We calculated the spatial extent of 22 MVGs in the National Vegetation Information System which are at risk from climate change (Fig. 2). Shapefiles of MVGs were overlaid on climate change risk predictions to extract values. We excluded nine MVG categories which collectively occur across 15% of Australia (i.e. Inland aquatic - freshwater, salt lakes, lagoons; Cleared, non-native vegetation, buildings; Naturally bare - sand, rock, claypan, mudflat; Mangroves; Regrowth, modified native vegetation; Sea and estuaries; Unclassified forest; Unclassified native vegetation; Unknown/no data).

## Supplementary information

Figure S1

Table S1

Table S2

## Data Availability

The derived data used in this analysis are available as supplementary materials. Code used in this study is available upon request.
