# Supplementary material for: Safety margins and adaptive capacity of vegetation to climate change
Source: Sci Rep. 2019 Jun 3;9:8241. doi: 10.1038/s41598-019-44483-x (PMC6547698; doi:10.1038/s41598-019-44483-x)
Supplement: Supplementary file 1 — Figure S1 [file 41598_2019_44483_MOESM1_ESM.pdf]

SUPPLEMENTARY MATERIAL

Safety margins and adaptive capacity of vegetation to climate change

Rachael V. Gallagher*, Stuart Allen & Ian J. Wright

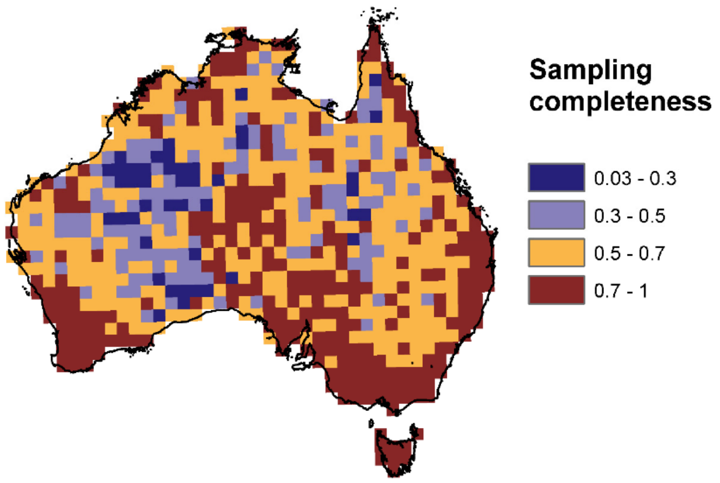

Figure S1. Sampling completeness of cleaned, vouchered herbarium specimens for the Australian flora estimated from the Chao1 species richness estimator. See Appendix S2 for values in each grid cell. Red areas (0.7-1) were included in analyses of spatial patterns of vegetation vulnerability.

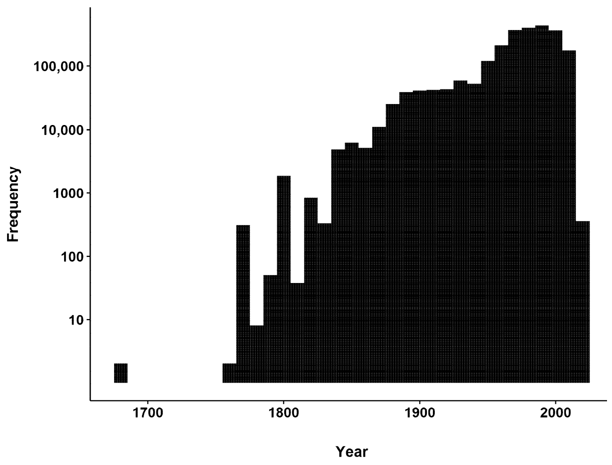

Figure S2. Frequency distribution of the year of collection of vouchered herbarium specimens analysed.
